# *Mycobacterium tuberculosis* and Dual* M. tuberculosis*/*M. bovis* Infection as the Cause of Tuberculosis in a Gorilla and a Lioness, Respectively, in Ibadan Zoo, Nigeria

**DOI:** 10.1155/2016/8568237

**Published:** 2016-05-15

**Authors:** Aina Adeogun, Olutayo Omobowale, Chiaka Owuamanam, Olugbenga Alaka, Victor Taiwo, Dick van Soolingen, Simeon Cadmus

**Affiliations:** ^1^Department of Zoology, University of Ibadan, Ibadan 200005, Nigeria; ^2^Department of Veterinary Medicine, University of Ibadan, Ibadan 200005, Nigeria; ^3^Zoological Garden, University of Ibadan, Ibadan 200005, Nigeria; ^4^Department of Veterinary Pathology, University of Ibadan, Ibadan 200005, Nigeria; ^5^Department of Pulmonary Diseases and Department of Clinical Microbiology, Radboud University, Nijmegen Medical Centre, Nijmegen, Netherlands; ^6^Diagnostic Laboratory for Bacteriology and Parasitology (BPD), Center for Infectious Disease Research, Diagnostics and Perinatal Screening (IDS), National Institute for Public Health and the Environment (RIVM), P.O. Box 1, 3720 BA Bilthoven, Netherlands; ^7^Tuberculosis and Brucellosis Research Laboratories, Department of Veterinary Public Health & Preventive Medicine, University of Ibadan, Ibadan 200005, Nigeria

## Abstract

Tuberculosis (TB) in zoo animals is an important public health problem in places where it occurs. This is even very important in countries where there is little public health awareness about the disease; thus confined animals in the zoo can be infected directly or indirectly by infected humans and vice versa. In Nigeria, the problem of TB is a major concern among both humans and cattle. Here, we present cases of* Mycobacterium tuberculosis* and* M. tuberculosis* ***/*** *M. bovis* infections in a female gorilla and a lioness, respectively, in a zoo in Ibadan, Nigeria. These cases were confirmed after bacteriological examinations and DNA from granulomatous lesions of the animals' carcasses were subjected to the Hain and spoligotyping techniques. Our findings reveal the first documented report of TB infections in a gorilla and a lioness in zoo animals in Nigeria. The public health risks of tuberculosis in zoological settings are therefore reemphasized.

## 1. Introduction

Tuberculosis (TB) remains a major public health problem globally [1]. The disease affects humans and other wide range species of nonhuman primates, elephants, carnivores, marine mammals, giraffes, rhinoceroses, buffaloes, and psittacine birds in different countries of the world including USA, Thailand, Sweden [2–6]. Globally, TB is mainly caused by* Mycobacterium tuberculosis* in humans and* M. tuberculosis* is one of the seven species constituting the* M. tuberculosis* complex (MTC) which includes* M. bovis,* a major pathogen of cattle causing bovine tuberculosis (BTB). Reports of TB infections in gorillas and members of the lion family are scarce in Nigeria despite the huge burden of the disease in the human population [1] and the endemicity of BTB in cattle [7, 8].

## 2. Case Report

Between 2009 and 2010, we investigated the death of a female gorilla and a lioness due to TB in Zoological Garden in Ibadan, Ibadan, southwestern Nigeria.

### 2.1. Case 1

A female lowland gorilla (*Gorilla gorilla*) of about 47 years of age was presented dead at the postmortem unit of the Department of Veterinary Pathology, University of Ibadan. The carcass was markedly emaciated with bony projections of the rib. The ocular and oral mucous membranes were moderately pale. There were several cream coloured firm nodules of varying sizes (5 mm–2 cm) in multiple organs including the lungs, liver, spleen, and the serosa. Microscopic examination of these nodules revealed typical granulomatous inflammation in affected organs characterised by caseous necrosis surrounded by zone of inflammation comprising macrophages, lymphocytes, plasma cells, and occasional giant cells. Based on the above, tentative diagnoses of generalized TB and uterine leiomyoma (fibroid) were made at the Department of Veterinary Pathology, University of Ibadan, Nigeria.

Specimens from the lungs and other affected tissues and organs with miliary nodules were decontaminated and digested as previously described by Cadmus et al. [9] using NALC- (N-acetyl-L-cysteine-) NaOH and followed by DNA extraction. Briefly, decontaminated samples were centrifuged for 10 minutes at 5 000 rmp and the supernatant was removed. 200 *μ*L of InstaGene matrix was added to the pellet and incubated and mixed in a thermomixer at 56°C for 30 min and later vortexed for 10 seconds. The product was again incubated and mixed at 99°C for 30 minutes and spun down for 3 minutes at 12 000 rmp. Finally, 20 *μ*L of supernatant from the resulting supernatant was aliquotted to carry out PCR reaction for spoligotyping and the Hain test as previously described [10, 11].

**Figure 1:**
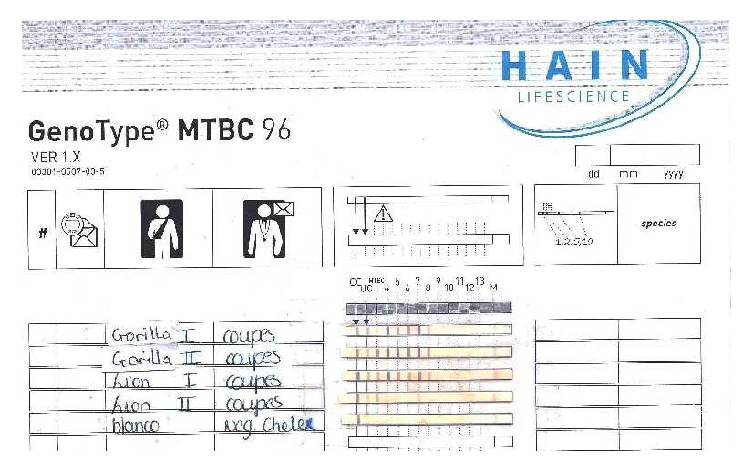
The result of the Hain test for the gorilla and the lioness.

Our result showed that only the Hain test confirmed* M. tuberculosis* (Figure 1) as the incriminating agent of the infection in the gorilla while the spoligotyping failed.

### 2.2. Case 2

**Figure 2:**
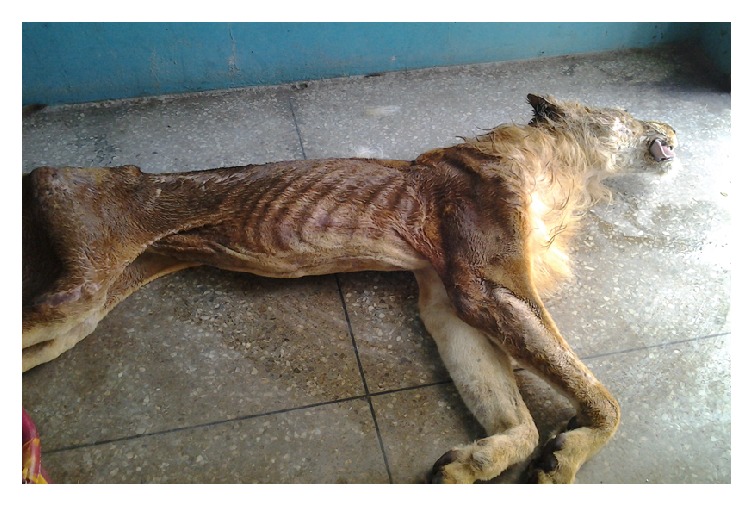
Emaciated lioness.

**Figure 3:**
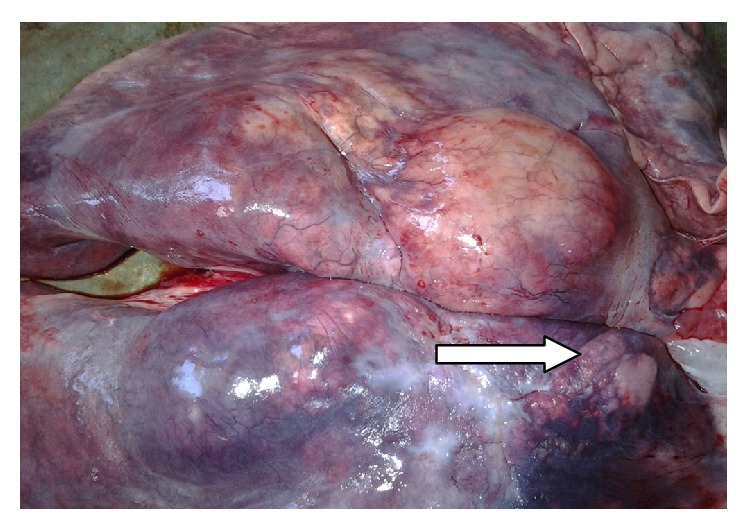
Photograph of lioness lungs showing multiple solitary and coalescing nodules of varying sizes (arrow).

**Figure 4:**
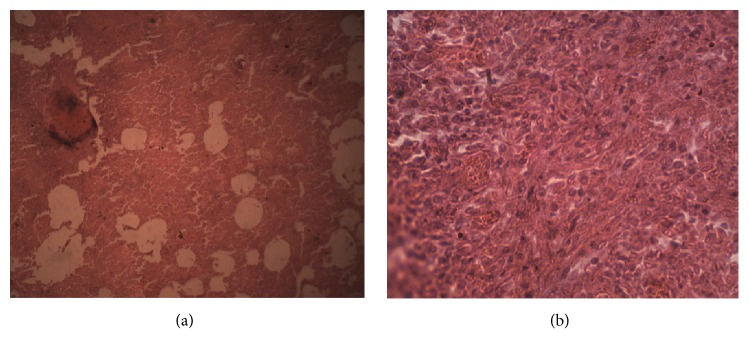
The micrograph in section (a) shows the lioness lungs with extensive alveolar collapse and multiple foci of granulomatous reactions in the lungs, ×100 H&E. (b) Higher magnification of section (a) showing extensive fibrosis, marked alveolar collapse, and mononuclear cellular infiltrations.

A female lion (*Panthera leo*) of about 15 years of age was presented at the postmortem unit of the Department of Veterinary Pathology, University of Ibadan. Postmortem examination revealed a markedly emaciated carcass with sunken eyes and pale mucous membrabes (Figure 2). The trachea and bronchi contained thick brownish mucopurulent froth. The lungs were diffusedly hyperemic with some localized foci of ecchymotic heamorrhages on the ventral surfaces of the left caudal and right middle lobes. Both lungs were consolidated and firm in consistency with nodules ranging from 0.5 to 2 cm in diameter spread throughout the parenchyma. Many of the nodules were solitary, while a few were confluent (Figure 3). Upon incision, the large nodules were observed to have abscesses in the caudal lobe of the right lung, while others were firm to hard with cheesy core. Some of the associated lymph nodes, particularly the pharyngeal and mediastinal, were edematous and enlarged. The liver, spleen, and kidneys were markedly congested and enlarged. Tentative morphological diagnoses were marked dehydration and emaciation; pneumonia; granulomatous, chronic active, and severe lymphadenopathy. Histopathology results revealed alveolar spaces containing infiltration of neutrophils and macrophages mixed with fibrin and extensive alveolar collapse with multiple foci of granulomatous reactions (Figure 4).

Specimens from the lungs and pharyngeal and mediastinal lymph nodes with miliary nodules were decontaminated and digested and DNA extraction was carried out as described for the gorilla above.

**Figure 5:**
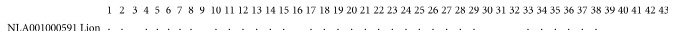
*M. bovis* spoligotype recovered from a lioness in a zoo in Ibadan, Nigeria.

The results of the Hain test (Figure 1) and spoligotyping technique (Figure 5) revealed the presence of* M. tuberculosis* and* M. bovis* from the lungs and mediastinal lymph nodes, respectively.

## 3. Discussion

We report the isolation of* M. tuberculosis* and* M. tuberculosis*/*M. bovis* in a gorilla and a lioness, respectively, in a private zoo in Ibadan, southwestern Nigeria. Tuberculosis caused by* M. tuberculosis* and* M. bovis* has been identified in a wide range of species, including nonhuman primates, elephants, and other exotic ungulates, carnivores, marine mammals, and psittacine birds [2, 3]. Disease associated with* M. tuberculosis* has occurred mostly within captive settings and does not appear to occur naturally in free-living mammals.* Mycobacterium tuberculosis* associated disease mostly occurs within captive settings and rarely appears naturally in free-living mammals [3]. In Nigeria, several reports have been made concerning human and bovine TB [7, 9, 12–15]. Globally, Nigeria ranks 4th among the TB burdened nations [1]; coupled with this, BTB is endemic among farm and slaughtered animals [7–9, 14]. Due to the high prevalence of human pulmonary TB in Nigeria and observed poor hygienic habits of zookeepers as well as visitors, animals within the private zoo in Ibadan are therefore exposed to possible risk of TB infections from humans.

The gorilla at this zoo was particularly at grave risk of exposure to TB, given the multitude of people who went visiting her, since she was a center of attraction in the zoo. The fact that she also lived in the zoo for about 42 years (brought into the zoo in 1962 when she was about 5 years) also meant that old age and confinement might have contributed to her vulnerability and death to TB.

The lioness had dual infection resulting from* M. tuberculosis* and* M. bovis*. The* M. tuberculosis* infection could be due to similar scenario presented for the gorilla (particularly as it relates to occasional confinement during which the human contact is close and highest) and her relatively old age. The most likely source of her* M. bovis* infection could be due to contaminated raw meat she was fed, mainly from the abattoir where 4.3% prevalence of BTB has been reported in slaughtered cattle [16] and with reports of* M. bovis* infection in slaughtered goats [14]. The fact that the animals fed to the lions in the zoo are not subjected to prior postmortem checks makes them vulnerable to* M. bovis* infection.

From the public health perspective, since the zoo environment is mostly congested with human population at festive seasons, it becomes easy for animals and zookeepers to become infected by TB patients who in most instances may be unaware of their illnesses despite obvious symptoms due to limited public health awareness about the disease [17, 18]. Similar scenario has accounted for zoo animal infection in other settings in Sweden, Thailand, and USA, [3, 5, 6, 19].

In conclusion, this study confirms cases of TB due to* M. tuberculosis* and* M. tuberculosis*/*M. bovis* in a gorilla and a lioness, respectively, in a zoo in Nigeria. Due to the high prevalence of human and BTB in Nigeria, we advocate that more public health precautions be taken by zookeepers in the country and most TB endemic countries with high contact between humans and wildlife. In addition, efforts should be put in place to routinely screen zookeepers who can indirectly transmit infections from the visiting public to the animals. In the same vein, public contacts with the animals must be reduced to the barest minimum. More importantly, raw meat/animals fed to zoo animals should go through routine meat inspection checks in order to control infection with* M. bovis*. Finally, we advocate continuous public health awareness to zoo visitors as a way of stepping up TB enlightenment and control in disease endemic countries.
